# CT and MRI Findings of Autoimmune Polymorph Bifocal Pancreatitis Mimicking Pancreatic Adenocarcinoma: A Case Report and Review of the Literature

**DOI:** 10.1177/2324709615576988

**Published:** 2015-03-16

**Authors:** Roman Rotzinger, Hendrik Bläker, Marcus Bahra, Timm Denecke, Christian Grieser

**Affiliations:** 1 Charité—Universitätsmedizin Berlin, Berlin, Germany

**Keywords:** autoimmune pancreatitis, pancreatic adenocarcinoma, mimicking, bifocal, pancreatic cancer, AIP, CT, MRI

## Abstract

Autoimmune pancreatitis is a rare type of chronic pancreatitis. It is supposed to be a pancreatic manifestation of an immune-complex modulated systemic disorder. In contrast, pancreatic adenocarcinoma is the most frequent malignant neoplasm of the pancreas. Within the rare type of focal autoimmune pancreatitis, only few presentations with multifocal pancreatic lesions have been described. Herein we report a case of a 58-year-old patient with autoimmune pancreatitis presenting with bifocal manifestations of the pancreatic head and tail, mimicking pancreatic adenocarcinoma clinically, on computed tomography and magnetic resonance imaging. Typical imaging findings of autoimmune pancreatitis are compared with typical findings in pancreatic carcinoma. The diagnostic dilemma of differentiating between both entities is discussed. A review of the present literature regarding multifocal presence of autoimmune pancreatitis is performed.

## Introduction

Autoimmune pancreatitis (AIP) is a rare type of chronic pancreatitis (occurring between 5% and 6%).^1^ It subsumes various morphologic descriptions such as nonalcoholic duct-destructive, lymphoplasmatic sclerosing or duct-narrowing chronic pancreatitis.^1^ Common to all AIP is a pancreatic manifestation of an immune-complex modulated systemic disorder that is characterized by prominent lymphocyte infiltration and associated organ fibrosis.^1^ The inflammatory process characteristically results in diffuse swelling of the pancreatic parenchyma and may finally lead to organ dysfunction.^2^ However, a focal type of AIP has been described before.^2^ Within these rare focal manifestations, only a few presentations of AIP with multifocal lesions have been reported.^3^ AIP occurs predominantly in elderly males and usually responds dramatically well to steroid therapy.^4^ Involvement of other organs is commonly seen.^4^ In contrast, pancreatic adenocarcinoma is the most frequent malignant neoplasm of the pancreas, which is mostly located in the pancreatic head and therefore may lead to extrahepatic cholestasis.^5^ Liver metastases are common findings. Although abdominal pain, new-onset diabetes mellitus, and jaundice are common, patients oftentimes clinically present without any symptoms.^5^ Complete tumor resection is still the only potentially curative therapy option for patients with ductal adenocarcinoma of the pancreas; however, only a minority of patients is resectable and 5-year survival rates of more than 20% after resection are rare.^6^

As there is no reliable diagnostic serological marker for AIP and the approach to the pancreas for histological examination is generally difficult, imaging is essential for the differentiation between AIP and pancreatic adenocarcinoma.^4^ Radiological workup may include computed tomography (CT), magnetic resonance imaging (MRI), and, to visualize the pancreatobiliary tree, endoscopic retrograde cholangiopancreatography (ERCP).^4^ Also, magnetic resonance cholangiopancreatography (MRCP) has been discussed recently as a noninvasive alternative, as it is becoming more and more preferable to diagnostic ERCP.^4,7^

Here we present an unusual case of AIP in a patient with bifocal pancreatic manifestations mimicking multifocal pancreatic adenocarcinoma on CT and MRI. A review of the present literature regarding multifocal presence of AIP was performed.

## Case Presentation

A 58-year-old female patient with a suspicious mass of the pancreas found in an ultrasound examination was referred to our hospital for further evaluation and treatment. She presented with pain radiating around her back, 3 days of nausea and vomiting, jaundice, and new-onset type 2 diabetes. The patient has had lost 10 kg bodyweight over the last 6 months unintendedly. She had no significant family history. The patient was a nonsmoker, and no alcohol or drug abuse was reported. Physical examination did not reveal any pathologic findings except of jaundice.

Laboratory tests revealed high levels of blood glucose (360 mmol/L). Inflammation biomarkers were low to normal by slightly lowered white blood cell count (4.18/nL) and normal C-reactive protein levels (0.49 mg/dL). Serum lipase levels were normal (34 U/L), serum amylase levels were not determined. Alkaline phosphatase was elevated (175 U/L), as well as liver enzymes (γ-glutamyltransferase, 152 U/L; total bilirubin, 8.8 mg/dL; alanine aminotransferase, 331 U/L; aspartate aminotransferase, 156 U/L). Tumor markers revealed elevated levels of CA 19-9 (100 U/L) but normal levels of CEA (1.7 µg/L) and AFP (3.6 µg/L). Serum calcium levels, ANA, or IgG4 levels were not determined.

**Figure 1.**
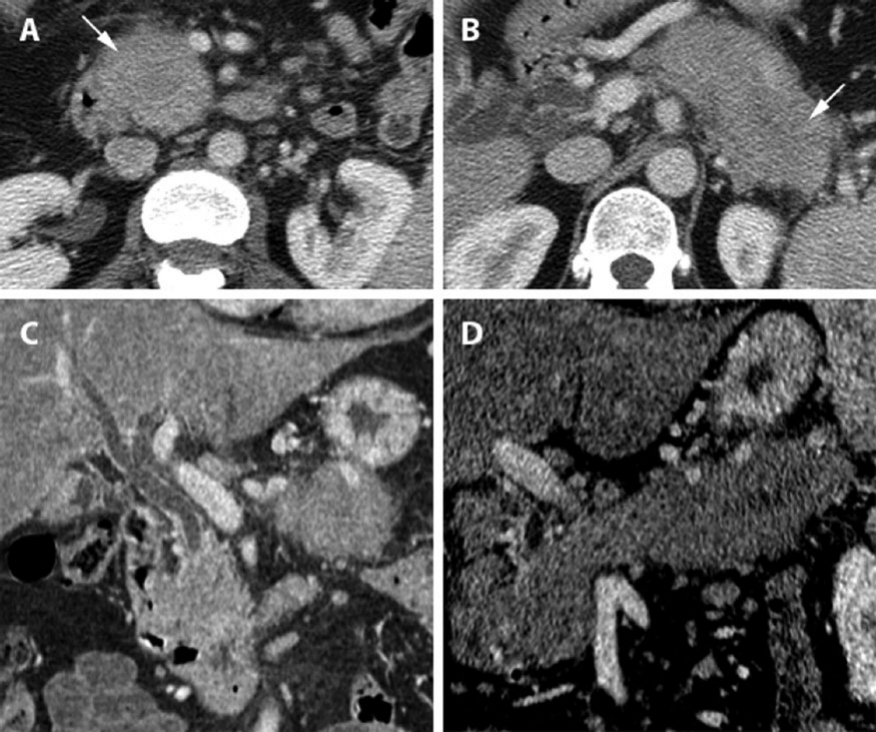
A 58-year-old female with multifocal autoimmune pancreatitis. The pancreas appears grossly enlarged. (A) Axial contrast-enhanced CT (venous phase) shows poorly enhancing focal lesions in the pancreatic head (arrow) and (B) tail (arrow). (C) Coronal contrast-enhanced CT shows intrahepatic cholestasis by stenosis of the bile duct. (D) CT curved reconstruction along the pancreatic duct shows both lesions, interruption and dilatation of the intrapancreatic tract.

On intravenously contrast enhanced CT the pancreas showed a focally well-defined, hypoenhancing mass in the pancreatic head and a diffusely swollen, hypoenhancing tail with ill-defined outlines due to stranding of the surrounding fatty tissue (Figure 1A and B). Intrahepatic cholestasis by stenosis of the bile duct was present (Figure 1C); however, the pancreatic main duct was not enlarged on CT. Locoregional lymphadenopathy was present (Figure 1A and B).

**Figure 2.**
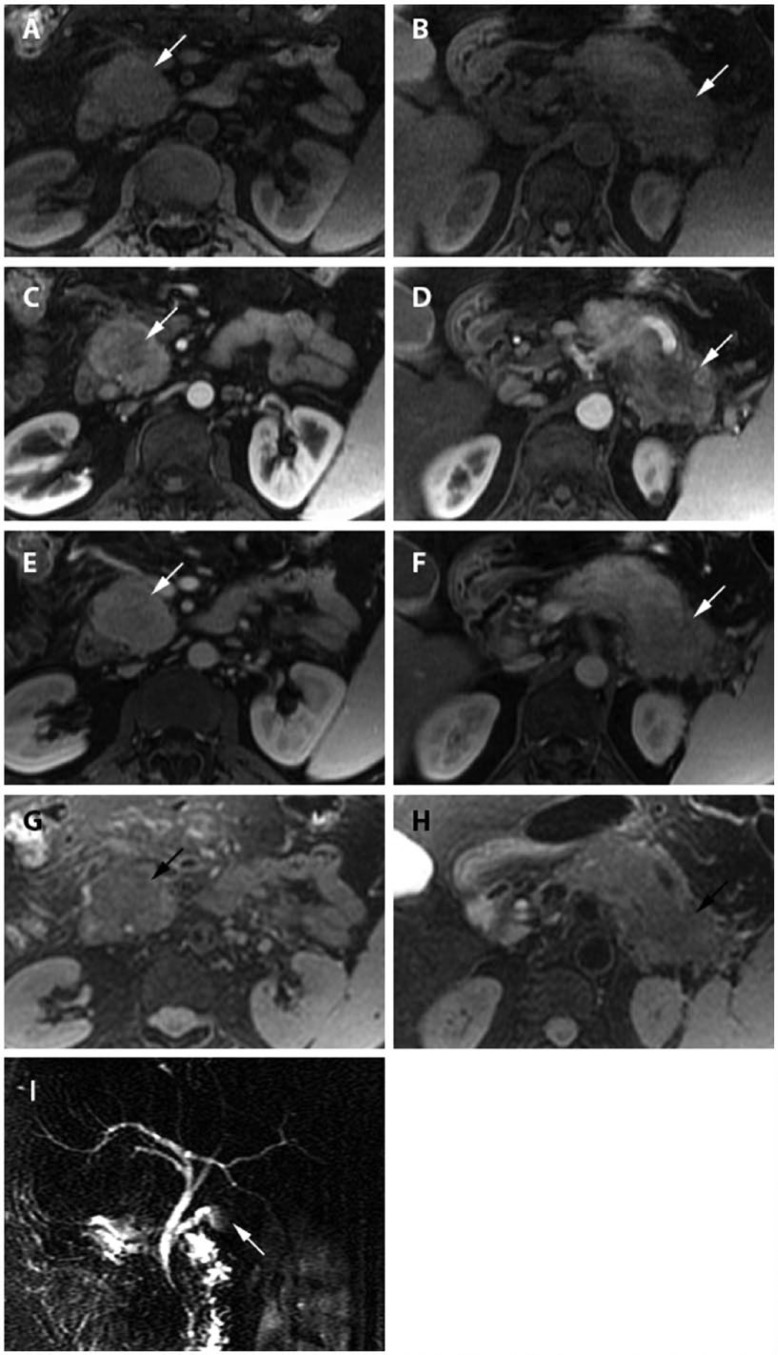
A 58-year-old female with multifocal autoimmune pancreatitis. The pancreas appears overall to be edematously swollen. (A) Axial T1-weighted MRI (LAVA) shows focal lesions in the pancreatic head (arrow) and (B) tail (arrow). (C, D) Corresponding axial contrast-enhanced T1-weighted MRI during arterial phase, (E, F) during portal venous phase, and (G, H) axial fat-saturated T2-weighted MRI during venous phase. (I) MRCP shows dilation and interruption of the pancreatic duct (arrow).

For further evaluation, MRI including MRCP was performed. In correlation to CT, MRI consistently revealed 2 focally well-circumscribed masses of the pancreatic head and tail. On native, fat-saturated, T1-weighted images (LAVA), the 2 solid lesions presented hypo- to iso-intensely (Figure 2A and B) as on native, fat-saturated, T2-weighted images (Figure 2G and H). Also on contrast-enhanced series the lesions presented hypo- to iso-intensely during arterial (Figure 2C and D) and portal venous phase (Figure 2E and F). They trended more toward iso-intensity in the venous phase, most likely by washout of contrast media (not shown). The MRCP showed stenosis of the bile duct (DHC), intrahepatic cholestasis, and hence dilatation of the DHC as well as dilatation and interruption of the pancreatic duct (Figure 2I).

Overall the suspicion of pancreatic adenocarcinoma leading to accompanying pancreatitis arose. Given the local respectability assessed on CT and MRI, a total pancreatectomy was chosen primarily in a curative intent to remove the mass together with the inflammatory tail in the diabetic patient.

A combined pancreatectomy, splenectomy, duodenectomy, and bilio-digestive anastomosis were performed. The peri- and postoperative course proved to be uncomplicated.

**Figure 3.**
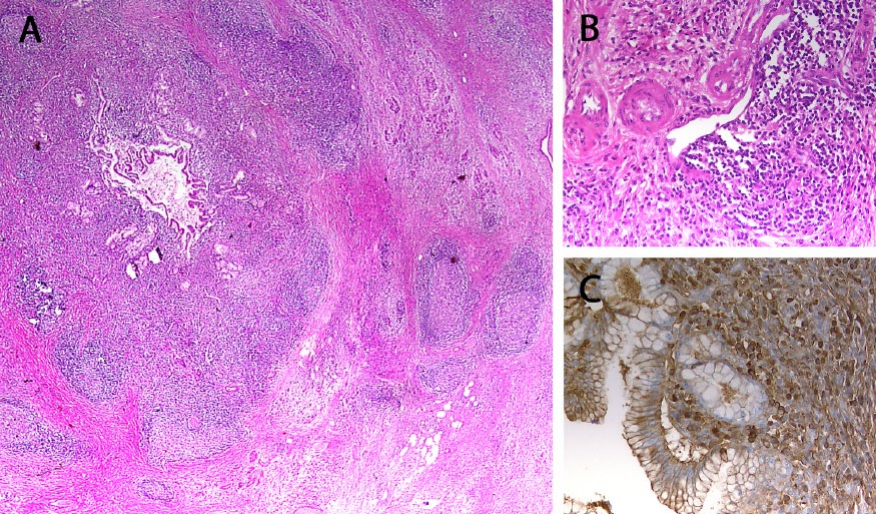
A 58-year-old female with multifocal autoimmune pancreatitis. Histopathologic evaluation shows dense inflammatory infiltrations composed of plasma cells and lymphocytes. (A) Large pancreatic ducts, bile ducts, and (B) intra- and extrapancreatic veins and nerves in the focus of inflammation. (C) Immunohistochemistry for IgG4 revealed an elevated concentration of IgG4-positive plasma cells.

On histopathologic evaluation, dense inflammatory infiltrations composed of plasma cells and lymphocytes were found. Large pancreatic ducts, bile ducts (Figure 3A), as well as intra- and extrapancreatic veins (Figure 3B) and nerves were in the focus of inflammation. Associated with chronic inflammation, pancreatic ducts displayed concentric fibrosis without granulocytic epithelial lesions (GELs). Immunohistochemistry for IgG4 (Figure 3C) revealed an elevated concentration of IgG4-positive plasma cells. Overall, no tumor was detected. A final diagnosis of autoimmune pancreatitis (AIP, type 1) was made.

## Discussion

Imaging is essential for the diagnosis of both AIP and pancreatic adenocarcinoma. Pancreatic adenocarcinoma usually presents as poorly enhancing mass on CT and MRI. Due to its common location in the pancreatic head, it oftentimes leads to cholestasis. Irregular narrowing and stenosis of the pancreatic duct on ERCP or MRCP is characteristic. A low-density rim on CT, which is hypointense on T2-weighted MRI, is typical but not necessary, as a loss of fatty lobulation and peripancreatic stranding.^8,9^ Although AIP usually presents as a diffusely enlarged pancreas, a focal type has been described.^2^ Within these cases only a few multifocal presentations have been reported.^3^

Against this background, it is oftentimes difficult to distinguish between AIP and pancreatic adenocarcinoma radiologically. Both entities frequently have to be diagnosed based on careful consideration of a combination of typical clinical, radiological, serological, and histopathological features.^10^ However, sometimes none of the above characteristics may solve the diagnostic dilemma. In practice, it is generally difficult to obtain adequate biopsy material for histopathological examination on endoscopic ultrasound- or CT-guided biopsy. Small sample size gained by core biopsy or fine needle aspiration may often either be false negative or necessitate laparatomy and surgical resection.^11^ Inflammation biomarkers are usually unspecific. Elevated tumor biomarkers can commonly be seen in patients with AIP,^4^ as elevated serum levels of IgG4 were reported in up to 10% of patients with pancreatic adenocarcinoma.^12^

In the demonstrated case, all imaging studies (CT, MRI, and MRCP) were highly suspicious for pancreatic adenocarcinoma with accompanying pancreatitis of the upstream organ partition. Standard laboratory parameters (such as inflammation biomarkers and tumor biomarkers) were consistent with imaging findings. Admittedly serum levels of IgG4 were not determined; however, elevated levels could not have ruled out a clinically and radiologically suspicion of malignancy as sometimes IgG4 is falsely positive in subjects without AIP including pancreatic carcinoma (up to 10%).^4,12^ Calculated steroid therapy could have been diagnostic for AIP, yet it should only be performed in patients with highly suggestive AIP after thorough workup for pancreatic adenocarcinoma. Hence, it was not indicated in this case. Moreover, steroid therapy usually takes weeks to months to show improvement.^5^ Against the background that imaging, clinical, and laboratory findings were typical and concordant, we reasonably assumed a diagnosis of pancreatic adenocarcinoma and did not perform core biopsy.^13^ Especially given that our patient had the rare opportunity to undergo surgical resection as the only curative therapy of pancreatic adenocarcinoma, it was supportable to suggest surgery as first-line treatment not to miss the short therapeutic window of curative surgical resection.^6^

## Conclusion

We demonstrated an unusual case of AIP with multifocal polymorphic manifestations mimicking pancreatic adenocarcinoma and accompanying pancreatitis on CT and MRI. AIP must always be considered as a differential diagnosis for pancreatic adenocarcinoma by careful assessment of clinical, radiological, serological, and histopathological features. Nevertheless, we argue some patients will never clearly meet these criteria. In particular, cases of misdiagnosed multifocal AIP run the risk of unnecessary surgery for presumed pancreatic adenocarcinoma.
